# Menstrual hygiene access and practices in Latin America: scoping review

**DOI:** 10.1590/1518-8345.6736.4029

**Published:** 2023-10-23

**Authors:** Viviane Caroline de Oliveira, Érica Dumont Pena, Gisele Nepomuceno de Andrade, Mariana Santos Felisbino-Mendes

**Affiliations:** 1 Universidade Federal de Minas Gerais, Escola de Enfermagem, Belo Horizonte, MG, Brasil.

**Keywords:** Health Knowledge, Attitudes, Menstrual Hygiene Products, Menstruation, Public Health Nursing, Reproductive Health, Review, Conocimientos, Actitudes y Práctica en Salud, Productos para la Higiene Menstrual, Menstruación, Enfermería en Salud Pública, Salud Reproductiva, Revisión, Atitudes e Prática em Saúde, Produtos de Higiene Menstrual, Menstruação, Enfermagem em Saúde Pública, Saúde Reprodutiva, Revisão

## Abstract

**Objective:**

to synthesize available evidence related to menstrual hygiene access and practices in Latin America and the Caribbean.

**Method:**

literature scoping review with research protocol registered in the Open Science Framework, carried out in the bibliographic databases: PubMed, Scopus, Web of Science and Portal Regional da Biblioteca Virtual em Saúde. Data were analyzed using simple descriptive statistics and thematic analysis.

**Results:**

15 publications were included, the majority of which addressed adolescents in Brazil: 12 articles, two technical reports and a course conclusion monograph. As recurring themes in the publications, the following stand out: Access to dignified conditions for managing menstrual hygiene; Need for access to information on menstrual hygiene management; and Practices for managing menstrual hygiene.

**Conclusion:**

adolescents report difficulties in accessing toilets, water and absorbent materials, and lack of information about menstrual health, including in schools, leading to school absenteeism. Thus, gaps in the Latin American scientific literature reveal inequalities and diversity in menstrual experiences intersected by categories such as gender, social class and ethnicity

## Abstract (es)

**Objetivo:**

sintetizar la evidencia disponible relacionada con el acceso y las prácticas de higiene menstrual en América Latina y el Caribe.

**Método:**

revisión de alcance de la literatura con protocolo de investigación registrado en el Open Science Framework, realizada en las bases de datos bibliográficas: PubMed, Scopus, Web of Science y Portal Regional da Biblioteca Virtual em Saúde. Los datos fueron analizados mediante estadística descriptiva simple y análisis temático.

**Resultados:**

se incluyeron 15 publicaciones, la mayoría de las cuales trataban sobre adolescentes en Brasil: 12 artículos, dos informes técnicos y una monografía de trabajo de conclusión de curso. Como temas recurrentes en las publicaciones se destacan: acceso a condiciones dignas para el manejo de la higiene menstrual; necesidad de acceso a información sobre el manejo de la higiene menstrual; y prácticas para el manejo de la higiene menstrual.

**Conclusión:**

adolescentes informan dificultades para acceder a baños, agua y materiales absorbentes, y falta de información sobre la salud menstrual, incluso en las escuelas, lo que lleva al ausentismo escolar. De esta manera, las lagunas en la literatura científica latinoamericana revelan desigualdades y diversidad en las experiencias menstruales interseccionadas por categorías como género, clase social y etnia.

## Abstract (pt)

**Objetivo:**

sintetizar evidências disponíveis relacionadas ao acesso e práticas de higiene menstrual na América Latina e Caribe.

**Método:**

revisão de escopo da literatura com protocolo de pesquisa registrado no Open Science Framework, realizada nas bases de dados bibliográficas: PubMed, Scopus, Web of Science e Portal Regional da Biblioteca Virtual em Saúde. Os dados foram analisados por estatística descritiva simples e análise temática.

**Resultados:**

foram incluídas 15 publicações, cuja maioria abordava adolescentes no Brasil: 12 artigos, dois relatórios técnicos e uma monografia de trabalho de conclusão de curso. Como temas recorrentes nas publicações, destacam-se: acesso a condições dignas para o manejo da higiene menstrual; necessidade de acesso à informação sobre manejo da higiene menstrual; e práticas para manejo da higiene menstrual.

**Conclusão:**

adolescentes relatam dificuldades de acesso a sanitários, água e materiais absorventes, e falta de informação sobre saúde menstrual, inclusive nas escolas, levando ao absenteísmo escolar. Assim, lacunas na literatura científica latino-americana revelam desigualdades e diversidade nas experiências menstruais interseccionadas por categorias como gênero, classe social e etnia.

**Highlights:**

**(1)** Lack of access to hygiene products, toilets and water for personal hygiene.

**(2)** Invisibility of the problem of period poverty in Latin America.

**(3)** Lack of physical structure in schools aggravates and leads to school absenteeism.

**(4)** Menstrual health literacy was insufficient for the demands of adolescents.

**(5)** Primary studies on menstrual hygiene in Latin America are scarce.

## Introduction

Menstrual health can be understood as a complete state of physical, mental and social well-being in relation to the menstrual cycle ^(^ 1 ^)^. Having menstrual health can have different meanings, depending on the lifestyle of people who menstruate. In general, they are related to access to information, supports - material and symbolic - to take care of their bodies according to their particular needs, diagnosis, treatment and appropriate care for discomforts and disorders related to the menstrual cycle, and positive contexts, respectful and free from exclusion, discrimination, coercion or violence ^(^ 1 ^)^. 

Several cultures have stigmas and beliefs related to menstruation that result in negative repercussions, such as the lack of dialogue on the subject ^(^ 2 ^)^, and the difficulty in accessing information about the physiology of menstruation, body care and accessible and appropriate practices to absorb or collect menstrual blood ^(^ 3 ^)^. Therefore, access to menstrual health education by the young population could contribute to the deconstruction of these stigmas and discrimination, in addition to facilitating the identification of diseases, such as dysmenorrhea and endometriosis ^(^ 4 ^)^. However, the literacy of this population has been shown to be inadequate and insufficient to meet the demand of young women in the global scenario ^(^ 5 ^)^. 

This study will address one of the aspects of menstrual health, menstrual hygiene management (MHM), defined as “access to information and education on ways to manage menstruation, clean materials to absorb and/or collect menstrual blood, and privacy to change them as many times as necessary”, including access to clean water and soap for body hygiene ^(^ 6 ^-^ 7 ^)^. The lack of these inputs has been conceptualized as period poverty, a term that refers to barriers (financial, social, cultural and political) in accessing products for menstrual hygiene, sexuality education, including information on menstrual management, free of taboos, and access to health services ^(^ 6 ^)^. It is estimated that globally more than 500 million people who menstruate do not have access to MHM ^(^ 5 ^)^. 

Recently, menstruation management has been more widely discussed as a public health issue on the global stage, especially in lower middle income countries (LMICs ^(^ 6 ^,^ 8 ^)^. In these settings, for example, the lack of water, sanitation and hygiene (WASH) affects spheres of people’s daily lives ^(^ 9 ^)^ and is responsible, in part, for period poverty, as already noted and documented in African ^(^ 10 ^-^ 11 ^)^ and Asian ^(^ 12 ^-^ 13 ^)^ countries. However, few studies have investigated the topic in Latin America and the Caribbean, where sanitation problems are also challenging ^(^ 14 ^)^. In addition to those factors, historical negligence related to menstrual health, silence and social etiquette marked by discretion on the subject in all spaces ^(^ 6 ^)^, as well as androcentrism ^(^ 3 ^)^, could also partly explain the unmet needs and the scarcity of studies on the subject in the region. 

Therefore, it can be inferred that MHM is an unattended aspect of sexual and reproductive health (SRH), especially in settings of greater social vulnerability such as LMICs, constituting an additional challenge for adolescents and women, as well as other aspects of SRH have been neglected in these contexts, such as teenage pregnancy ^(^ 15 ^)^, abortion ^(^ 16 ^)^ and contraception ^(^ 17 ^)^. In addition, the Latin American scenario is marked by political, economic and social inequalities, and identifying and mapping studies that seek to investigate MHM and the impacts related to period poverty can contribute to the discussion in society, as well as allow the construction of more effective measures related to SRH, mainly related to menstrual health. In 2019, there was a global call and few countries in the Americas, for example, established actions in favor of menstrual health, such as Mexico, the United States of America and Brazil ^(^ 5 ^)^. With that, the methodology of this study was selected because it allows a broader view of this complex and expanding subject, and mainly because it allows the identification and analysis of possible gaps in knowledge. 

Therefore, considering that menstrual hygiene is increasingly recognized as a public health issue that is closely related to the fulfillment of the United Nations (UN) Sustainable Development Goals (SDGs ^(^ 7 ^,^ 18 ^)^, we sought to synthesize the available evidence related to access and practices of menstrual hygiene in Latin America and the Caribbean (LAC) and, with that, contribute to the visibility and dimension of this basic human need of women, which lack is configured in a public health problem called period poverty. 

## Method

### Type of study

This is a systematic review, of the scoping review type, with a research protocol registered in the Open Science Framework ( https://osf.io/eg3pu/). This study was developed and structured according to the recommendations of the JBI ^(^ 19 ^)^ and the checklist Preferred Reporting Items for Systematic Reviews and Meta-Analyses extension for Scoping Reviews (PRISMA-Scr) ^(^ 20 ^)^. We comply with the Sex and Gender Equity in Research (SAGER) guidelines ^(^ 21 ^)^, which indicate the careful use of sex and gender in scientific publications and the standardization of these definitions. In this study, we used gender identities “girl” and “woman” and “female” as a search category. Although female gender, assigned at birth, may include transmasculine gender identities, these identities were not highlighted in the search. 

### Information sources

A bibliographic survey was carried out in the following databases: PubMed, Scopus, Web of Science and Portal Regional da Biblioteca Virtual em Saúde. 

### Population

The study population consisted of 1,981 publications found in searches carried out in the databases and from the references of documents read integrally.

### Eligibility criteria

Articles from peer-reviewed journals and grey literature (not peer-reviewed), including online reports, published in scientific journals, and book chapters available online, were considered eligible. To be included, studies and documents should present: primary data on menstrual hygiene management practices or access to menstrual hygiene management; portray people who live in Latin American and Caribbean countries; documents in English, Portuguese and Spanish, published between January 2011 and July 2022.

It was considered that the specific descriptor “Menstrual Hygiene Products” was introduced to Medical Subject Headings (MeSH) in 2007, and that until 2010 there were no records of publications in the PubMed database that met the eligibility criteria.

### Study variables

The variables collected from each study were: year of publication, country of origin, affiliation of the authors, authors’ education, indexed journal, type of publication, methodological design, characteristics of the population, sample size, and the results found on the menstrual hygiene access and practices.

### Selection of evidence sources and data extraction process

The extraction of data from the scientific production analyzed in this study was performed using an instrument adapted from the JBI form ^(^ 19 ^)^, prepared by the researchers in the Microsoft Excel 2016 ^®^ program. 

For the search and selection of studies, we used the mnemonic combination PCC ^(^ 19 ^,^ 22 ^)^: P Population - People of the female gender (women, girls, teenagers); C Concept 1 - Access to menstrual hygiene; period poverty; C Concept 2 - Menstrual hygiene practices; C Context - Countries of Latin America and the Caribbean. Thus, the following guiding question was established: “How are menstrual hygiene access and practices for adolescent girls and women in Latin America and the Caribbean?”. 

Initially, for the recognition of the descriptors, a non-systematized search was carried out in databases and, with this, an overview of the knowledge produced on the subject was obtained. In addition, it was possible to identify the index terms used to describe the topic under study, which contributed to outline the search strategy of this research more specifically.

The searches were carried out in August 2022, through remote access to the databases, from the registration in the journal portal of the Coordenação de Aperfeiçoamento de Pessoal de Nível Superior (CAPES), via Comunidade Acadêmica Federada (CAFe), with the Universidade Federal de Minas Gerais (UFMG) login. 

The results obtained in the bases were exported to the Rayyan reference manager, developed by the Qatar Computing Research Institute (QCRI) ^(^ 23 ^)^, for the removal of duplicate documents, selection and screening of studies, by two researchers independently and blindly. Using a previously prepared guide for the selection of studies and in the event of disagreements, the researchers discussed until consensus. The documents that met the inclusion criteria were analyzed by reading the manuscripts integrally. Finally, manual searches were performed in the references of the included studies. 

### Search strategy

The construction of the search strategy was carried out using Boolean operators AND and OR, descriptors included in the MeSH of the United States National Library of Medicine and in Health Sciences Descriptors (DeCS), in addition to some free terms, namely : Puberty, Girl, Teenager, Woman, Female, Menarche, Menstruation, “Menstrual Cycle”, Pads, “Menstrual Hygiene Products”, “Menstrual Hygiene”, “Menstrual Health”, “Period Poverty”, “Menstrual Pads”, “Vaginal Tampon”, “Menstrual Hygiene Management”, “Health Education”, “Sexual Education”, “Health Knowledge, Attitudes and Practice”, Access, Practice, “Health-Related Behavior”, “Health Attitudes and Practice”, “Health Behavior”, and their counterparts in Spanish and Portuguese.

### Summary of results

Initially, the data were analyzed using simple descriptive statistics of the main characteristics of the included documents. In addition, a thematic analysis ^(^ 24 ^-^ 25 ^)^ of the included documents was carried out, involving six steps: 1. Familiarization with data; 2. Initial encoding generation; 3. Search for themes; 4. Review of themes; 5. Definition of themes; and 6. Writing. These steps were carried out from discussions between the researchers and the codification of themes, which was based on the literature on menstrual health. The categories were selected by observing their recurrence, and because they are capable of covering the conceptual universe of the studies and the large groups of themes that they describe. 

### Ethical aspects

This study used data available from public domain. Therefore, there was no need to submit the study to the Research Ethics Committee.

## Results

**Figure 1 -:**
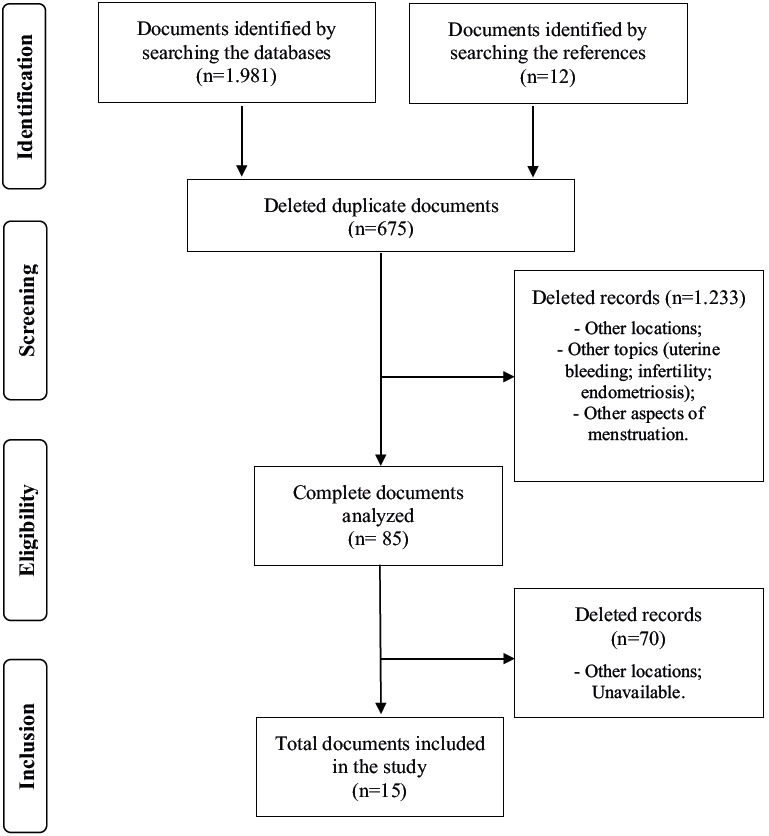
Flowchart of the study selection process. Belo Horizonte, MG, Brazil, 2022

1,993 articles were selected for screening, 1,981 retrieved through the search and 12 identified through the search in the references. Of the total, 247 publications were identified in Web of Science, 493 in Scopus, 396 in PubMed and 845 in Biblioteca Virtual de Saúde. After excluding duplicate publications (n= 675), 1,306 remained for the first selection stage (reading titles and abstracts). A total of 85 publications were selected for the second selection stage (complete reading of the texts), remaining at the end 15 publications that integrated the review ( Figure 1). 

Of the 15 publications included in this review, 10 were published in the last 5 years. In 12 of them, the authors were linked to higher education institutions, and in 3 the authors were linked to a non-governmental organization, namely: United Nations Children’s Fund (UNICEF). Fourteen main authors were identified among the analyzed publications, with their education (graduation) in: biological sciences (n=3), psychology (n=2), anthropology/sociology (n=2), nursing (n=2), medicine (n=2), physiotherapy (n=1), occupational therapy (n=1), and economics (n=1).

Regarding the methodology used in the studies, 60.0% (n=9) adopted a quantitative methodological approach, in which data were collected through questionnaires and analyzed with the aid of statistics. Of these, cross-sectional studies predominated (n=5), followed by cohort studies (n=4). Most studies were conducted in Brazil (n=7, 46.7%), with adolescents (n=8, 53.3%) and adult women (n=6, 40.0%), and published in international journals, exclusively in English (n=9, 60.0%). The characteristics of the studies are presented in Figure 2. 

**Figure 2 –:** Characteristics of the studies that were part of the scoping review. Belo Horizonte, MG, Brazil, 2022

Country/Year of Publication	Study Design	Participants	Age Group	Main Findings
Mexico/2012 ^(^ 26 ^)^	Cross-sectional	405 teenagers	12 to 15 years old	Access to information
Bolivia and Argentina/2013 ^(^ 14 ^)^	Mixed (systematic/cross-sectional review)	21 Basic Sanitation managers	NI‡ Access to MHM*	
Brazil/2013 ^(^ 27 ^)^	Cohort	307 women	18 to 45 years old	Menstrual hygiene practices
Bolivia/2013 ^(^ 28 ^)^	Qualitative	157 people (teenagers, mothers, teachers)	NI‡ Access to information and MHM*	
Mexico/2014 ^(^ 29 ^)^	Cross-sectional	602 teenage girls	11 to 16 years old	Access to information and MHM*
Colombia/2017 ^(^ 30 ^)^	Mixed (cross-sectional/qualitative)	246 teenage girls	14 to 15 years old	Access to information and MHM*
Haiti/2017 ^(^ 31 ^)^	Cohort	101 women and 49 college students	18 to 24 years old (College students)	MHM access and practices*
Belize/2017 ^(^ 32 ^)^	Mixed (cross-sectional/qualitative)	429 families and 246 women	15 to 49 years old	Access to MHM*
Brazil/2018 ^(^ 33 ^)^	Qualitative	39 teenagers	12 to 17 years old	Access to MHM*
Brazil/2018 ^(^ 34 ^)^	Cross-sectional	66 women	17 to 43 years old	Menstrual hygiene practices
Brazil/2020 ^(^ 35 ^)^	Cohort	49 college students	19 to 30 years old	Menstrual hygiene practices
Brazil/2021 ^(^ 36 ^)^	Quantitative	118,628 teenagers	13 to 18 years old	Access to MHM*
Brazil/2021 ^(^ 37 ^)^	Cross-sectional	167 teenage girls	12 to 24 years old	Access to MHM*
Mexico/2022 ^(^ 38 ^)^	Cohort	193 teenage girls	5th and 6th grade†	Access to information
Brazil/2022 ^(^ 39 ^)^	Cross-sectional	177 women	18 to 49 years old	Access to MHM*

*MHM = Menstrual Hygiene Management; †5th and 6th grade = The study did not specify the age of participants; ‡NI = No Information (the studies do not specify the age of participants)

The scope of scientific production on access and practices of menstrual hygiene was analyzed and organized by observation of recurrence, that is, by similarity of content, emerging three categories: 1) Access to dignified conditions for MHM ^(^ 26 ^,^ 28 ^-^ 30 ^,^ 38 ^)^; 2) Need for access to information ^(^ 14 ^,^ 28 ^-^ 33 ^,^ 36 ^-^ 37 ^,^ 39 ^)^; and 3) Practices for MHM ^(^ 27 ^,^ 31 ^,^ 34 ^-^ 35 ^)^. The summary of the results identified by the studies in this review is presented in Figure 3. 

**Figure 3 –:** Themes of studies on menstrual hygiene in Latin America and the Caribbean. Belo Horizonte, MG, Brazil, 2022

Themes of studies on menstrual hygiene in Latin America and the Caribbean
Access to information for menstrual hygiene management
Lack of information about ways to manage menstruation ^(^ 29 ^-^ 30 ^)^; dialogue between adolescents and mothers provide positive attitudes towards MHM ^(^ 26 ^)^; interventions aimed at increasing knowledge about MHM ^(^ 38 ^)^; discomfort in seeking information and guidance on MHM ^(^ 28 ^)^.	
Access to dignified conditions for menstrual hygiene management
Difficulty accessing toilets, privacy, clean water and absorbent materials ^(^ 14 ^,^ 37 ^,^ 39 ^)^, including at school ^(^ 30 ^,^ 33 ^,^ 36 ^)^; experience of grief and embarrassment during the menstrual period related to difficulty with MHM ^(^ 29 ^)^^,^^(^ 37 ^)^^-^^(^ 39 ^)^ and school absenteeism ^(^ 28 ^,^ 30 ^,^ 36 ^)^; need for public policies to guarantee dignity for MHM ^(^ 31 ^)^; sufficient condition for MHM ^(^ 32 ^)^.	
Menstrual hygiene practices
Segregation of women during the menstrual period ^(^ 31 ^)^, use of disposable pads for MHM ^(^ 34 ^)^, no association between the use of sanitary pads and the presence of vaginosis or candidiasis ^(^ 27 ^)^, use of menstrual cups for MHM ^(^ 34 ^)^^-^^(^ 35 ^)^.	

^*^MHM = Menstrual Hygiene Management

## Discussion

This scoping review showed the existence of a small number of studies and documents about access and practices of menstrual hygiene in LAC. The findings reinforce the invisibility of the public health problem that is period poverty in the region, and point to an important gap in the scientific literature on the subject, indicating insufficient delimitation of issues related to MHM and, consequently, difficulty in recognizing this problem in society in all areas. In addition, it corroborates the scenario of vulnerability of women and adolescent girls living in LAC.

The results of the documents analyzed in this review corroborate those of other studies in which the lack of physical structure for MHM in schools is evident, mainly in LMICs ^(^ 12 ^,^ 40 ^-^ 43 ^)^, such as: lack of clean water and soap to wash hands, products for MHM, such as disposable pads and often even toilet paper, in addition to privacy. They also highlight the lack of basic sanitation and the negative impact on issues of daily life, education, health and well-being ^(^ 14 ^,^ 31 ^)^. These are basic inputs for a dignified life, which historically have been problems faced by LAC countries ^(^ 43 ^)^. 

The findings of studies on access to information showed that adolescent girls who talked to their mothers ^(^ 26 ^,^ 30 ^)^ and who received information in schools ^(^ 38 ^)^ were considered more likely to feel prepared to experience menstruation. They were also those who related menstruation to a positive experience ^(^ 26 ^)^. Thus, access to information and knowledge about menstruation and ways of managing it constitute a fundamental and essential human right for quality of life and health outcomes ^(^ 4 ^)^. However, studies do not really address the quality of information that adolescent girls receive from family and/or school, as they are often not prepared to meet the needs of girls. Added to this is the evidence that menstrual health literacy has been shown to be insufficient and unable to meet the demands of adolescents living in low, middle and high income countries ^(^ 4 ^)^. 

The difficulty of accessing menstrual hygiene products was also shown in the studies, but incipiently in LAC. It is known that in low- and middle-income countries women and girls cannot choose the product or material they wish to manage their menstruation. In these contexts, families often report not being able to afford basic hygiene items and, therefore, the disposable pad is considered an item of difficult access ^(^ 44 ^-^ 47 ^)^. This review found only two studies that address this issue in LAC ^(^ 37 ^,^ 39 ^)^, carried out with immigrant women and adolescents living in Brazil. It is known that for women of reproductive age, menstrual hygiene products are an important part of life, and the lack of these perpetuates negative experiences and unhygienic practices related to menstruation, with repercussions on their daily lives and human dignity. Studies show, for example, that period poverty is associated with a higher occurrence of sexual and urinary infections, in addition to issues in the field of mental health, related to stigma, taboo, fear, exclusion and shame of menstruation ^(^ 5 ^)^. Likewise, it is associated with school absenteeism, as reported by some studies in LAC regions ^(^ 5 ^,^ 28 ^,^ 30 ^,^ 36 ^)^, a problem also faced in other LMICs ^(^ 42 ^)^, which may further increase gender inequality and contribute to perpetuate the cycle of poverty. 

There are several products available on the market for MHM, with varying degrees of quality, accessibility and acceptability. However, this study recognizes a limited view of the range of practices used to manage menstruation, because they were poorly reported in the identified studies ^(^ 27 ^,^ 34 ^-^ 35 ^)^. These studies point out that disposable pads seem to be the most used for MHM, which can be explained by their wide availability in different commercial points, and because they are provided free of charge by some governmental programs and non-governmental organizations (NGOs). However, this review did not find studies with people who reported using homemade materials (cotton fabric) or improvised materials (paper towel, toilet paper or others) for MHM, as evidenced by studies conducted in African ^(^ 9 ^,^ 43 ^)^, South Asian ^(^ 48 ^-^ 49 ^)^ and North American ^(^ 12 ^,^ 50 ^)^ countries. Therefore, these materials cannot be discarded as practices of people living in rural communities or in low-income environments, the reality of many countries in LAC ^(^ 5 ^)^. Likewise, some subgroups that are more socially vulnerable and that probably experience period poverty were also not represented in the documents, such as people deprived of liberty or homeless, reinforcing the invisibility of the problem and of women in the region. Thus, future studies and research on menstrual health in the region should consider the investigation of these unsafe practices and vulnerable population groups, and could also seek to measure the cost of period poverty for society. 

The findings of this review indicate that access to information and dignified conditions for MHM are essential for the menstrual health of people who menstruate. Therefore, it is crucial to understand menstrual health as a tool for health promotion, and its promotion as something that also contributes to achieving gender equality ^(^ 3 ^)^ and increasing the population’s literacy on the subject ^(^ 4 ^)^, which in turn reinforces the important role of health care and education in achieving this. It is known that nurses are professionals who assist women in all stages of life, including the school environment, in which nursing care is related to providing items for MHM ^(^ 51 ^)^, carrying out actions of health education, promoting access to information about the physiology of puberty ^(^ 52 ^)^ and contributing to the creation of facilitating school environments that support adolescents to experience the menstrual period with dignity ^(^ 40 ^)^. These actions can benefit people who menstruate and also constitute windows of opportunity to promote other aspects in the field of reproductive health that may be equally neglected. 

A limitation of this scoping review is the non-use of specific terms to include the transgender public in the search strategy. However, if articles were found that addressed this theme, this would not be considered an exclusion criterion. This group may have been underrepresented in the study.

Methodological limitations were identified in the body of evidence. Most studies relied primarily on self-reported measures of exposure or outcome ^(^ 26 ^,^ 29 ^-^ 31 ^,^ 33 ^,^ 37 ^,^ 39 ^)^. Self-reported information on menstrual management and health outcomes is subject to reporting bias, as menstruation is taboo in most countries and participants may prefer not to answer questions about this topic, or feel embarrassed ^(^ 53 ^)^, which may constitute a conservative bias of underestimation of the identified access problems. However, self-reporting about menstruation, hygiene and other related aspects is essential for the development of studies on the subject, as it allows knowing the experience of individual and collective experience related to menstruation. 

## Conclusion

This scoping review enabled the mapping of published documents related to menstrual hygiene access and practices in LAC. The evidence found suggests that MHM is an understudied aspect in the field of SRH, and that women and adolescent girls in LAC experience significant challenges, especially when they suffer from lack of access to hygiene products, clean toilets with privacy and clean water for body and hand hygiene. Period poverty is a public health problem that has contributed to perpetuate negative experiences related to menstruation.

The results obtained in this review also contribute to reaffirming the role of nurses in addressing basic human needs, whose nursing care should be focused on the promotion and prevention of physical and emotional health individually and collectively for people who menstruate, in the field of SRH. This study demonstrated the existence of inequalities and diversity in menstrual experiences intersected by categories such as gender, social class and ethnicity, in addition to the scarcity of primary studies on MHM in LAC, reinforcing the invisibility of period poverty. Understanding the range of practices that women and adolescent girls use to manage menstruation makes it possible to envision ways and strategies for nursing care, and this is an indication of the need for more attention to the subject in society and science, in order to advance in understanding and in the complexity of the theme, closely related to meeting the SDG agenda.
